# Trajectory of bolt and length of plate in femoral neck system determine the stability of femur neck fracture and risk of subsequent subtrochanteric fracture : a finite element analysis

**DOI:** 10.1186/s12891-023-06579-4

**Published:** 2023-06-07

**Authors:** Chang-Ho Jung, Yonghan Cha, Jun Young Chung, Chan Ho Park, Tae Young Kim, Jun-Il Yoo, Jung-Taek Kim, Yongho Jeon

**Affiliations:** 1 Department of Mechanical Engineering, Ajou University, Suwon, Korea; 2 Department of Orthopaedic Surgery, Eulji university hospital, Daejeon, Korea; 3 Department of Orthopaedic Surgery, Ajou University School of Medicine, Ajou Medical Center, 164, World cup-ro, Yeongtong-gu, Suwon, 16499 Korea; 4 Department of Orthopaedic Surgery, New Daesung Hospital, Bucheon, Korea; 5 Department of Orthopedic Surgery, Inha University Hospital, Incheon, Korea

**Keywords:** Bolt trajectory, Femoral neck fractures, Fixation, Pauwels type III

## Abstract

**Background:**

This study aimed to analyze the differences in the stability of fractures, stress distribution around the distal-most screw according to the length of the plate and the trajectory of the bolt in Pauwels type III femoral neck fracture using the femoral neck system (FNS).

**Methods:**

Finite element models of Pauwels type III femoral neck fractures were established with surgical variations in the trajectory of the bolt (central, inferior, valgus, and varus) and length of the lateral plate (1- and 2-hole plate). The models were subsequently subjected to normal walking and stair-climbing loads.

**Results:**

The screw-holding cortical bone in subtrochanter in the model with a 2-hole plate and the bolt in the inferior trajectory and the models with 1-hole or 2-hole plate and the bolt in valgus trajectory had shown greater maximum principal strain than the models with central or varus trajectories. The gap and sliding distance on the fracture surface were larger with inferior or varus trajectories of the bolt and smaller with the valgus trajectory of the bolt under both loads, compared to those of the central trajectory.

**Conclusion:**

For the fixation of Pauwels type III femoral neck fracture, the trajectory of the FNS bolt and the length of the plate affect the mechanical stability of the fracture and the strain of cortical bone around the distal-most screw. The surgical target should stay on the central trajectory of the bolt and the 2-hole plate’s mechanical benefits did not exceed the risk.

## Introduction

The surgical strategies for the management of femoral neck fractures are either fixation or arthroplasty [1, 2]. A displaced fracture is associated with an increased risk of non-union and osteonecrosis in older patients in whom the durability of the artificial joint is expected to last longer than the expected life of the patient, which is an obvious indication for arthroplasty. However, fixation is the desired method of treatment for undisplaced fractures in young patients [3]. Although the diverse spectrum of clinical presentation with mixed properties complicates the decision-making process, fixation is the generally recommended treatment for undisplaced fractures even in geriatric patients [4].

Pauwels’ type III femoral neck fracture is characterized by a large vertically oriented fracture angle that maximizes shear force in combination with a vertically oriented weight load. Consequently, this type of fracture is often associated with high rates of fixation failure and nonunion [5]. Studies have reported a nonunion incidence of 16–59% and a femoral head necrosis incidence of 11–86% in unstable Pauwels type III femoral neck fractures [6]. As a result, orthopedic surgeons are continuously seeking better fixation methods and treatment strategies for Pauwels’ type III femoral neck fracture.

The most common methods for fixing femoral neck fractures are the multiple cancellous screw technique (MCS) and the dynamic hip screw (DHS). While the MCS provides torsional stability and reduces the risk of injury to the blood supply to the femoral head, it cannot prevent vertical shear displacement, leading to high failure rates when used for Pauwels’ type III fractures. Therefore, a fixed-angle device like the DHS is recommended for fixing Pauwels’ type III femoral neck fractures, although it is mainly used for extracapsular hip fractures [7–11]. Recently, femoral neck system (FNS, DePuy Synthes, Oberdorf, Switzerland) joined the armamentarium with superior results, as shown in biomechanical experiments with Pauwels’ type III fracture model [12].

Although FNS has a similar macrostructure to the DHS as it has an extramedullary plate with a fixed angular bolt that allows controlled sliding along the screw or bolt axis, FNS has several differences compared to the DHS [13]. Compared to DHS, FNS, which has a shorter lateral plate and an anti-rotation screw designed to enter through the bolt at a divergent angle, has minimized the required surgical exposure. The bolt of FNS has a smooth surface with a cylindrical structure that lacks the macrostructure to hold the trabecular bone of the femoral head. [14]

In contrast to the DHS, of which several specifics such as the position of the screw tip and the length of the lateral plate, were examined previously, the influence of surgical variations of the FNS such as the length of the lateral plate and trajectory of the bolt on the stability of the femoral neck fracture is yet to be evaluated.

The FNS has a “side plate and screw” structure similar to the DHS; however, the length of the lateral plate is extremely shorter compared to that of the DHS, and this provides surgeons with two options for the length of the lateral plate, 1-hole or 2-hole long. Previous studies reported that DHS with its short lateral plate was biomechanically incompatible with fracture fixation. [15, 16] Similarly, two types of FNS plates were compared in a study involving femoral neck fractures with a Pauwel angle of 70 degrees. The results showed that the 2-hole plate had less displacement than the 1-hole plate. However, the study’s credibility was later questioned due to an inappropriate definition of contact in the research methods. Therefore, the conclusion of the comparison remains unclear [12, 17].

The present study hypothesized that the length of the plate and the trajectory of the bolt may affect the stability of the fracture surface and integrity of cortical bone around the distal-most screw. Therefore, our study aimed to analyze the differences in the stability of the fracture and stress distribution around the distal-most screw according to the length of the plate and the trajectory of the bolt in the management of Pauwels’ type III femoral neck fractures using a finite element model.

## Methods

### Ethical review statement

The requirement for informed consent was waived, and the study protocol was approved by the institutional review board (IRB) of our hospital as the acquisition of computed tomography (CT) scans was part of routine care and the use of the images posed minimal risk of harm to the patient (AJIRB-MED-MDB-21-696).

### Implant model

After scanning the FNS three dimensionally in the stereo-lithography format using a 3D scanner (Rainbow Scanner Prime; Dentium, Seoul, Korea) and micro-CT (SkyScan1173; Bruker-CT, Belgium), the implant model was reverse engineered by comparing the 3D scan images using Solidworks 2019 (Dassault System, France) and NRecon (Bruker-CT, Belgium).

### Three-dimensional modeling of the femur

The osteoporotic femur model was established from CT scans of the femur. Briefly, a CT scan of an 82-year-old patient with a left intertrochanteric fracture was used for this analysis. The height and weight of the patient were 160 cm and 54 kg, respectively. The Materialise Interactive Medical Image Control System Research 22.0 (MIMICS; Materialise, Antwerp, Belgium) software was used to reconstruct 3D models of the unfractured right femur from the CT images.

### Fracture models

A model of Pauwels type III femoral neck fracture was established with virtual osteotomy using 3-Matic 14 (Materialise) [18]. The fracture plane was aligned 60° to the horizontal plane [19]. Complete anatomical reduction was assumed without a fracture gap.

### Coordinate system

The coordinate system of Bergmann et al. was established around the femur [20]. The origin was located at the center of the best-fitting sphere of the femoral head. The axis femoral diaphysis represented the z-axis, whereas the frontal plane was defined to include the z-axis and was parallel to the femoral neck axis. The x-axis was assigned to lie in the frontal plane and to be normal to the z-axis. The y-axis was normal to both x- and z-axes.

### Meshing

Each model was meshed using 10-node tetrahedral elements. The average number of nodes and elements of eight finite element models were 6,631,690 and 4,818,424, respectively. All the elements were < 1 mm in size.

### Implant positioning

**Fig. 1:**
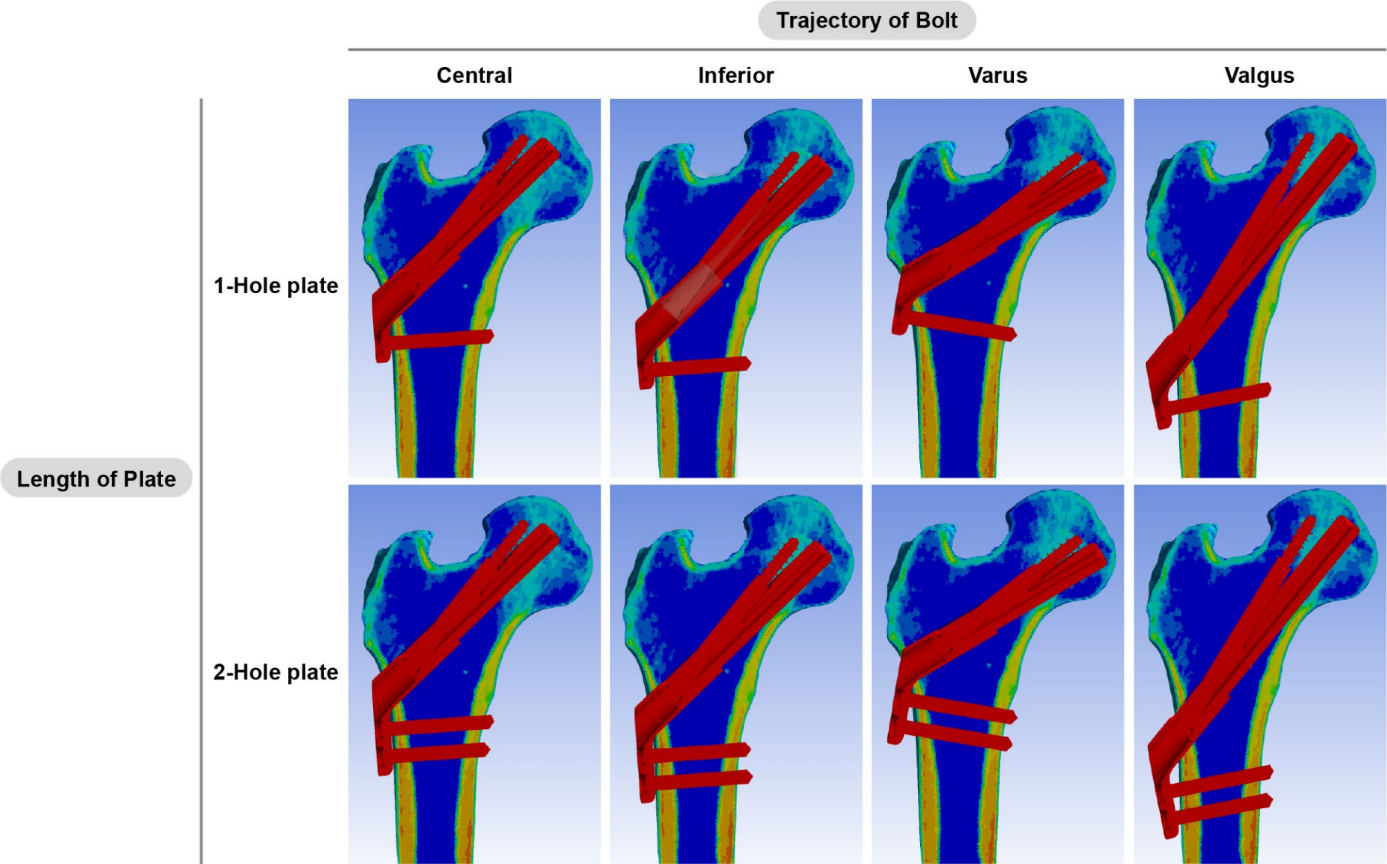
Pauwels type III femoral neck fracture finite element models with eight different combinations of surgical variations of the femoral neck system were established. The models in the upper row had a 1-hole plate and the models in the lower row had a 2-hole plate. The bolts were varied in the central, inferior, varus, and valgus trajectories from left to right

**Fig. 2:**
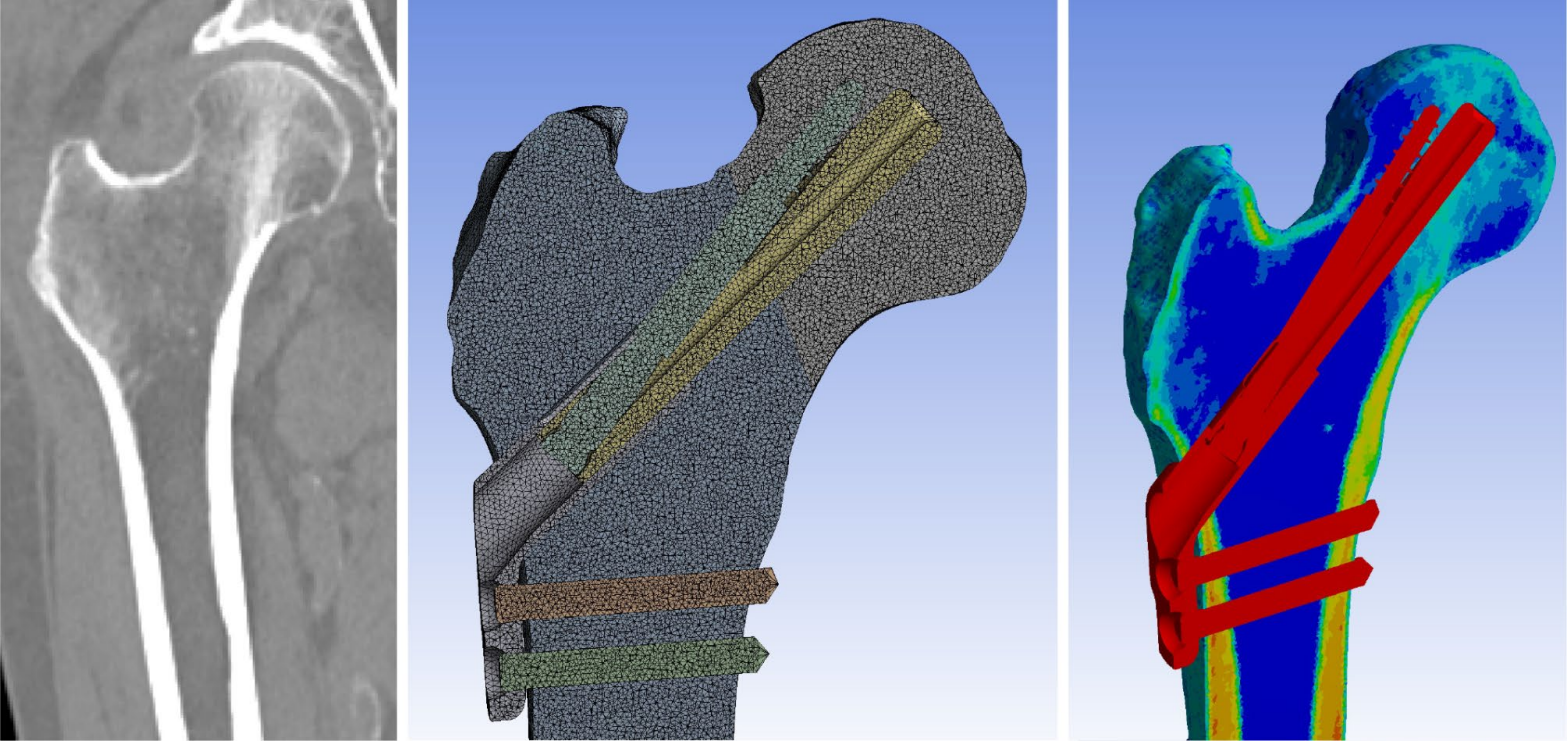
Each element of the femur was given the material property of the matched voxels of the computed tomography scan by calculating their Hounsfield units

**Fig. 3:**
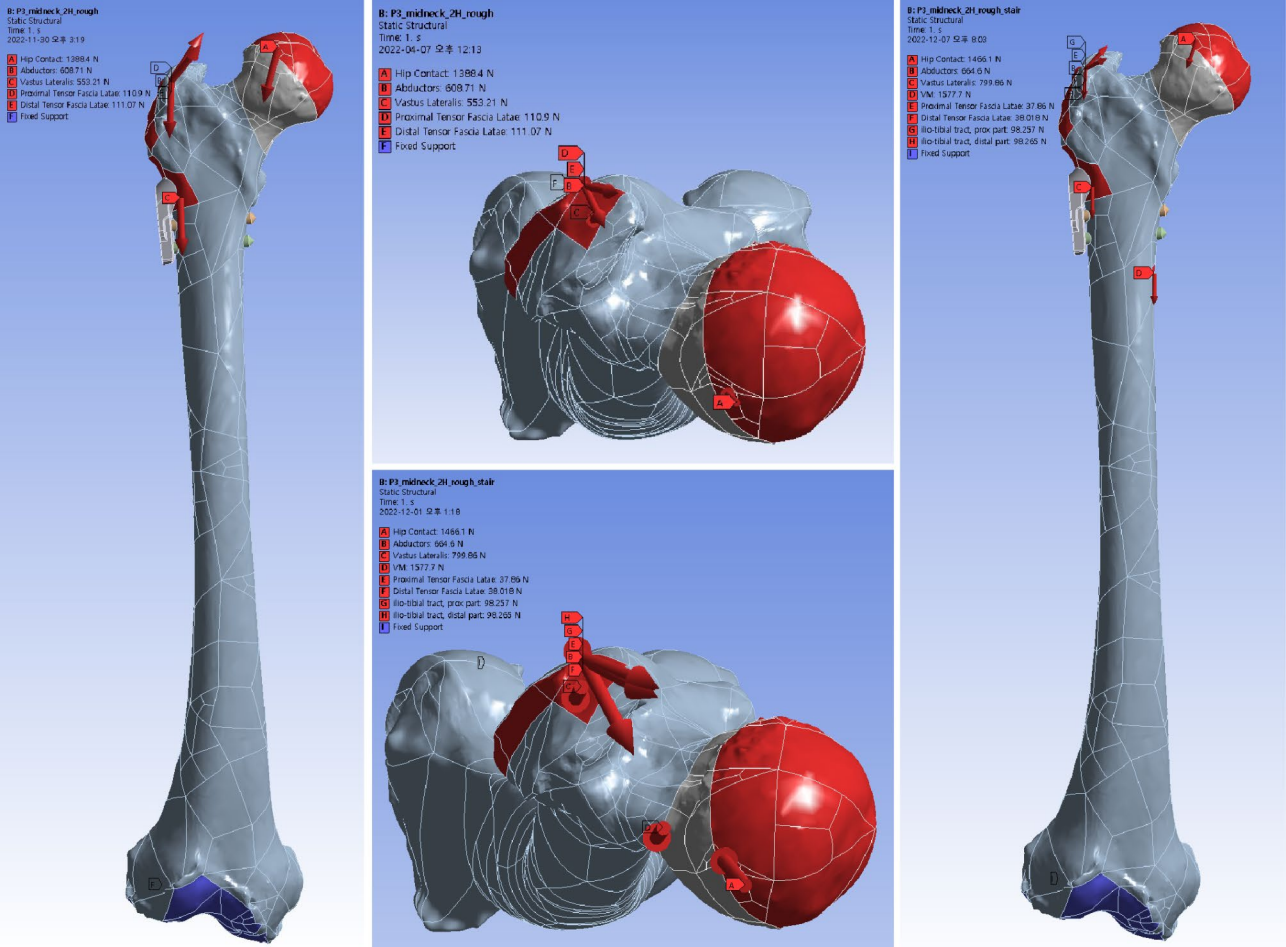
Fracture models were virtually loaded in the normal walking (A, B) and stair climbing (C, D) conditions. Weight load was transferred to the hemispheric surface of the femoral head at an inclination of 45° and retroversion of 25° in consideration of the abduction of the acetabulum and the combined anteversion of the acetabulum and femoral neck. The distal articular face of the femur was set to be fixed in the world coordinate system

Using the 3-Matic software, the 3D-fractured femur model was fixed with the 3D implant model. Eight different models were established, consisting of the standard central trajectory and three variations of the inferior, valgus, and varus trajectories, each with one or two-hole plates. The assembly of 90-mm long bolts and 90-mm long anti-rotation screws was virtually implanted in the central trajectory in the neck cortical corridor at a distance of 7 mm from the bolt tip and subchondral bone to establish central trajectory models (Fig. 1, 1st column). For models with the inferior trajectory, the trajectory of the bolt touched the endocortical bone, which was defined as 400 Hounsfield units (HU) (Figs. 1, 2nd column). The varus and valgus trajectories were angled by 15 degrees. (Figures 1 and 3rd and 4th column). The bolt lengths for each model were adjusted to keep the distance between the bolt tip and subchondral bone within the clinically relevant range of 5–10 mm [21] (Fig. 1). We utilised Boolean subtraction to replicate bone loss caused by the drilling and reaming procedure of FNS insertion to replicate the post-fixation construct. As the stability of the immediate postoperative condition determines long-term stability, the immediate postoperative period was assumed [14, 22].

### Solver

The present finite element analysis used the ANSYS 2019 R3 mechanical software (ANSYS Inc., Canonsburg, PA, USA) for solving (Fig. 2).

### Boundary conditions

The rough contact was assumed on the contact between the locking screws and plate and between the locking screws and femoral diaphysis. The interface between the bolt and anti-rotation screw was set as bonded. All other interfaces between the implant and the two fracture fragments were assumed to be frictional. The friction coefficients for bone-bone, bone-implant, and implant-implant interfaces were 0.46, 0.42, and 0.20, respectively [23]. The distal articular face of the femur was set to be fixed in the world coordinate system.

### Properties of the materials

Material properties of the bone elements were assigned using the mapping method proposed by Morgan et al. [24, 25] The mapping method encompasses matching the values from the CT HU to ash density, from ash density to apparent density, and from apparent bone density to Young’s modulus.^12^ The material properties of the bones were assigned into 150 groups (Fig. 2) [26, 27]. Bone elements with a mean HU of matching voxels over 400 were defined as cortical bone, while those with a mean HU of matching voxels were defined as trabecular bone [28].

The Poisson’s ratio of the bone elements was assumed to be 0.3 [24]. The material properties of the titanium alloy (Ti-6Al-7Nb) were given to the implants, elastic modulus, Poisson’s ratio, and yield strength, which were 105 GPa, 0.34, and 800 MPa, respectively [29]. The bone and implant were assumed to be isotropic and linear elastic materials.

### Loading condition

The loads of normal walking and stair climbing were assumed to each model, according to Bergmann et al.’s method [20, 23, 30]. A simplified loading condition for hip joint throughout walking and stair climbing cycles with simultaneous muscle forces of the hip abductor, tensor fascia latae, iliotibial band, vastus medialis and vastus lateralis were assigned to the origin or insertion site of respective muscles [23, 31]. (Table 1; Fig. 3)

**Table 1:** Load profile for normal walking and stair climbing (body weight = 54 kg) [31]

	x	y	z	Acting point
Walking				
Hip contact force	-315.4	-191.6	-1338.5	Articular contact surface of femoral head
Abductor	338.7	25.1	505.2	Greater trochanter
Tensor fascia latae, proximal	42.0	67.7	77.1	Greater trochanter
Tensor fascia latae, distal	-2.9	-4.1	-111.0	Greater trochanter
Vastus lateralis	-5.3	108.0	-542.5	Origin of vastus lateralis
Stair climbing				
Hip contact force	-346.3	-353.9	-1380.0	Articular contact surface of femoral head
Abductor	409.4	168.2	495.8	Greater trochanter
Ilio-tibial tract, proximal	61.3	17.5	74.8	Greater trochanter
Ilio-tibial tract, distal	-2.9	-4.7	-98.1	Greater trochanter
Tensor fascia latae, proximal	18.1	28.6	16.9	Greater trochanter
Tensor fascia latae, distal	-1.2	-1.8	-38.0	Greater trochanter
Vastus lateralis	-12.8	130.8	-789.0	Origin of vastus lateralis
Vastus medialis	-51.4	231.3	-1559.9	Origin of vastus medialis

*The forces (in Newtons) are given in the local coordinate system of the femur [20]

### Comparative parameters

The maximum and minimum principal strains were evaluated on the elements that belong to the femur and were compared to assess the risk of mechanical failure of bone. The von Mises stress (VMS) of the metal implants was evaluated to compare the risk of mechanical failure of implants.

The mechanical stability at the fracture interface was assessed with the interfragmentary gap and the sliding distance. Since the two surfaces of the fractured bone were in contact with each other, assuming a perfect reduction, the relative distance between each surface was calculated to determine the gap or sliding distance. Vertical movement relative to the surface is classified as gap distance, while parallel movement relative to the surface is classified as sliding distance. Microsoft Excel and Access (Microsoft, Redmond, WA, USA) were used to record the representative values. Less than 5% differences were considered as similar.

## Results

### Normal walking condition

**Fig. 4:**
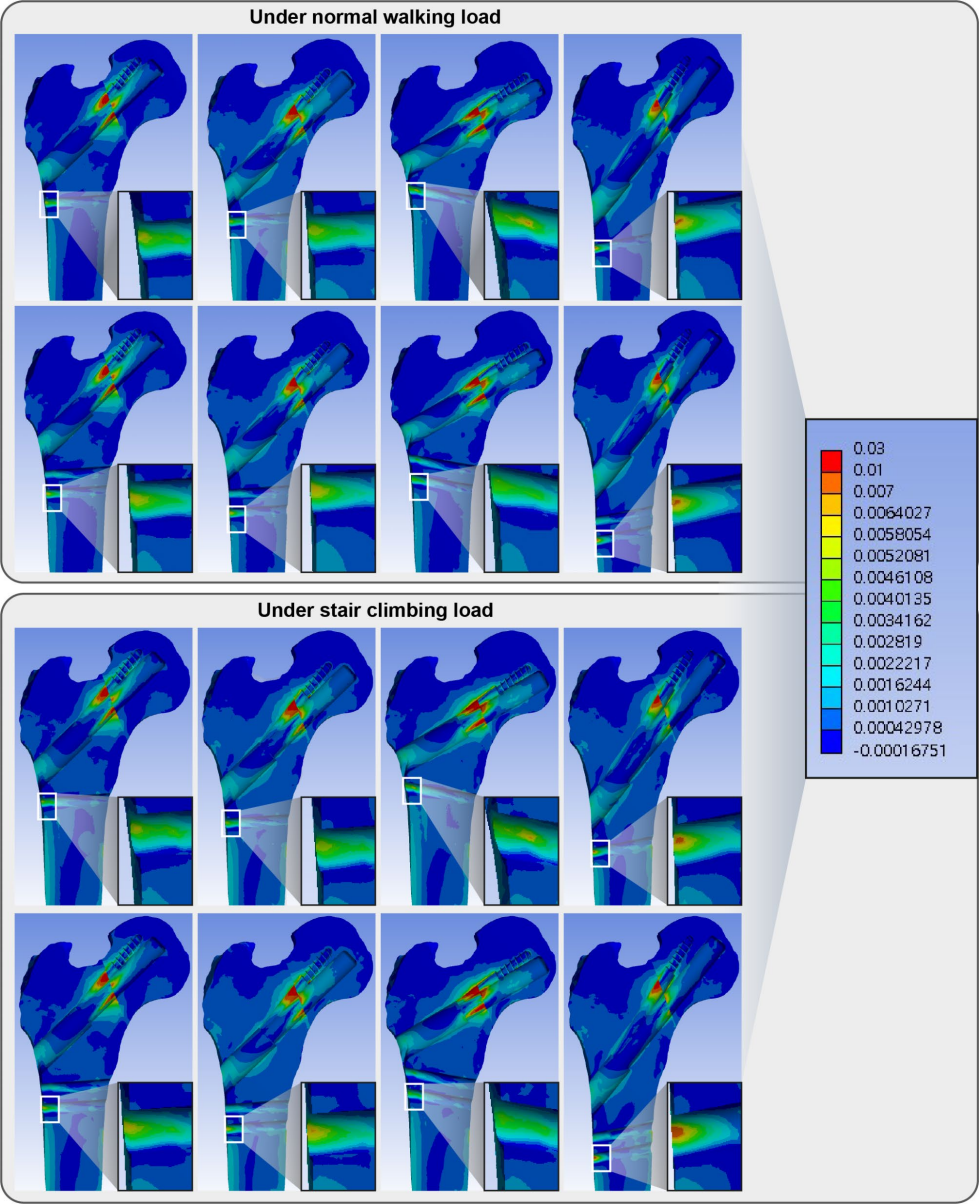
Band graphs depicting the maximum principal strain of the femur in the normal walking (upper eight graphs) and stair climbing (lower eight graphs) conditions. The graphs within each loading condition were arranged in the same sequence as in Fig. 1. All graphs share the color legend

**Fig. 5:**
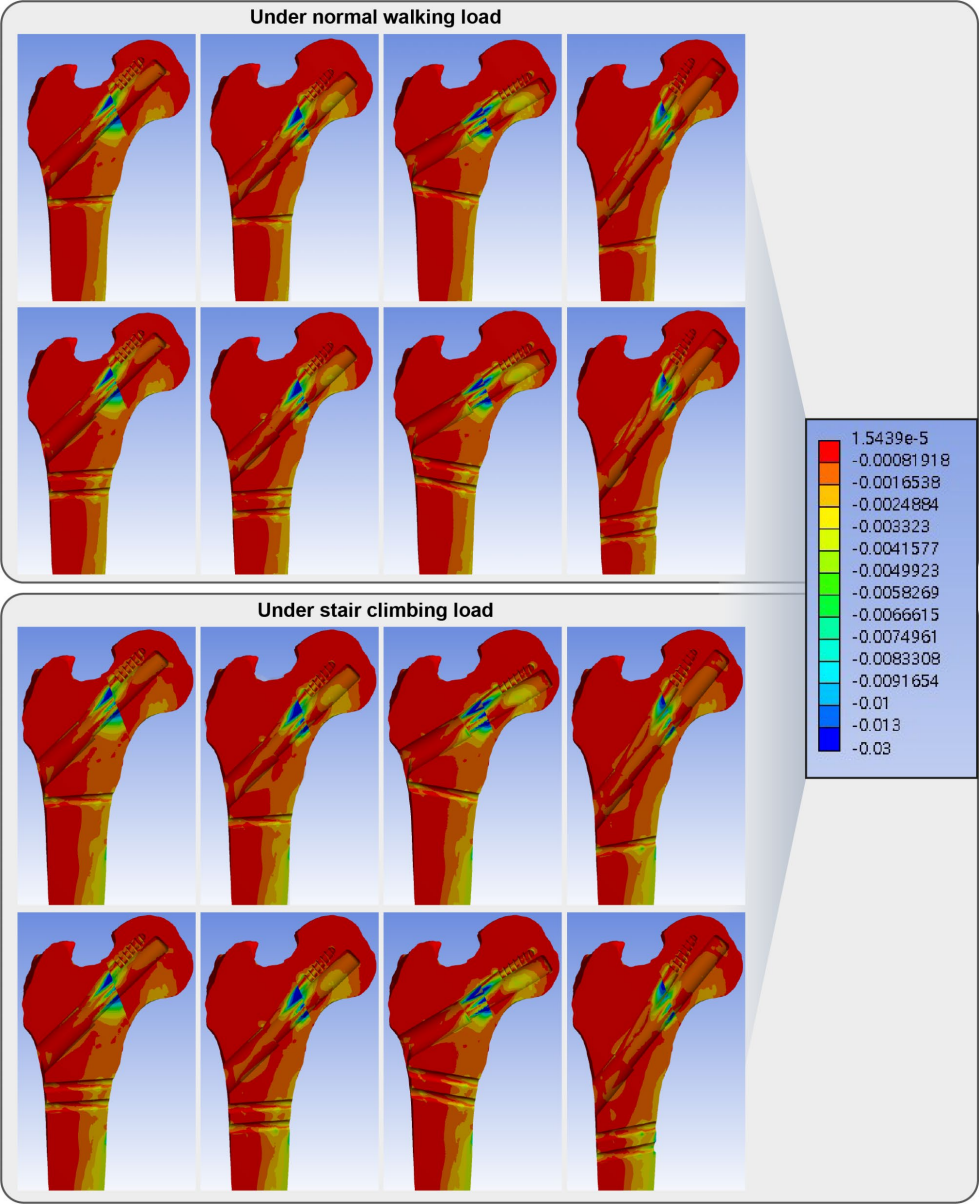
Band graphs depicting the minimum principal strain of the femur in the normal walking (upper eight graphs) and stair climbing (lower eight graphs) conditions. The graphs within each loading condition were arranged in the same sequence as in Fig. 1. All graphs share the color legend

The elements with maximum principal strain over 1% and minimum principal strain under − 1% indicated the trabecular bone located in the narrow cleft between the anti-rotation screw or under the plate which supports the bolt. (Figs. 4 and 5)

For cortical bone, the endosteal cortex in the inferior neck supporting the bolt in the models with bolts in the inferior trajectories had − 1.28% (model with the bolt in the inferior trajectory and 1-hole plate) and − 1.30% (model with the bolt in the inferior trajectory and 2-hole plate) of the peak minimum principal strain.(Fig. 5; Table 2) The screw-holding cortical bone distal to trochanter in models with a 2-hole plate and the bolt in the inferior trajectory, a 1-hole or 2-hole plate and the bolt in valgus trajectory had shown 1.44%, 1.11% and 1.15% of maximum principal strain, respectively. (Fig. 4; Table 3)

**Table 2:** Implant stress and stability at fracture interface according to the bolt trajectory and the length of plate

Load			Normal Walking					Stair Climbing						
Bolt trajectory			Center	Inferior	Varus	Valgus		Center		Inferior		Varus		Valgus
**Peak maximum principal strain**														
	1-hole plate													
	Cortical		0.81%	0.91%	0.69%	1.11%		0.74%		0.91%		0.66%		1.17%
	Trabecular		1.52%	1.35%	1.75%	1.14%		1.56%		1.47%		2.16%		1.23%
	2-hole plate													
	Cortical		0.96%	1.44%	0.92%	1.15%		1.03%		1.49%		0.92%		1.25%
	Trabecular		1.52%	1.44%	1.70%	1.20%		1.57%		1.51%		2.05%		1.35%
**Peak minimum principal strain**														
	1-hole plate													
	Cortical		-0.41%	-1.28%	-0.86%	-0.92%		-1.03%		-1.33%		-1.05%		-1.17%
	Trabecular		-2.27%	-2.24%	-3.57%	-1.63%		-2.19%		-2.36%		-4.19%		-1.72%
	2-hole plate													
	Cortical		-0.44%	-1.30%	-0.83%	-0.92%		-1.08%		-1.34%		-1.03%		-1.21%
	Trabecular		-2.36%	-2.20%	-3.63%	-1.65%		-2.23%		-2.34%		-1.19%		-1.48%
**von Mises stress on implant (MPa)**														
	1-hole plate		273.0	216.1	350.7	273.7		294.9		278.5		423.9		258.6
	2-hole plate		266.1	214.4	348.4	240.8		296.5		280.3		423.0		263.4
	Ratio (2-hole/1-hole)		97%	99%	99%	98%		101%		101%		100%		102%
**Fracture gap (mm)**														
	1-hole plate*		0.166	0.172 (104%)	0.199 (120%)	0.144 (87%)		0.178		0.177 (100%)		0.191 (107%)		0.154 (87%)
	2-hole plate*		0.160	0.168 (105%)	0.191 (120%)	0.140 (88%)		0.172		0.173 (100%)		0.185 (107%)		0.151 (87%)
	Ratio (2-hole/1-hole)		96%	97%	96%	98%		97%		98%		97%		98%
**Sliding distance between fracture fragments (mm)**														
	1-hole plate*		0.229	0.252 (110%)	0.417 (182%)	0.187 (82%)		0.208		0.2668 (128%)		0.500 (240%)		0.198 (95%)
	2-hole plate*		0.229	0.254 (111%)	0.411 (180%)	0.188 (82%)		0.208		0.268 (129%)		0.496 (238%)		0.199 (95%)
	Ratio (2-hole/1-hole)		100%	101%	99%	101%		100%		101%		99%		101%

* The percentage within parenthesis refers to the mechanical parameters corresponding to the trajectory column compared to those of central trajectory with the same plate length

**Table 3:** Maximum and minimum principal strain of cortical bone around the distal-most screw

Load		Normal walking					Stair climbing			
Bolt trajectory		Center	Inferior	Varus	Valgus		Center	Inferior	Varus	Valgus
Maximum principal strain of cortical bone										
1-hole plate		0.81%	0.91%	0.69%	1.11%		0.74%	0.91%	0.66%	1.17%
2-hole plate		0.96%	1.44%	0.92%	1.15%		1.03%	1.49%	0.92%	1.25%
Minimum principal strain of cortical bone										
1-hole plate		-0.41%	-0.46%	-0.49%	-0.50%		-0.43%	-0.46%	-0.48%	-0.93%
2-hole plate		-0.44%	-0.54%	-0.49%	-0.53%		-0.45%	-0.60%	-0.50%	-1.21%

**Fig. 6:**
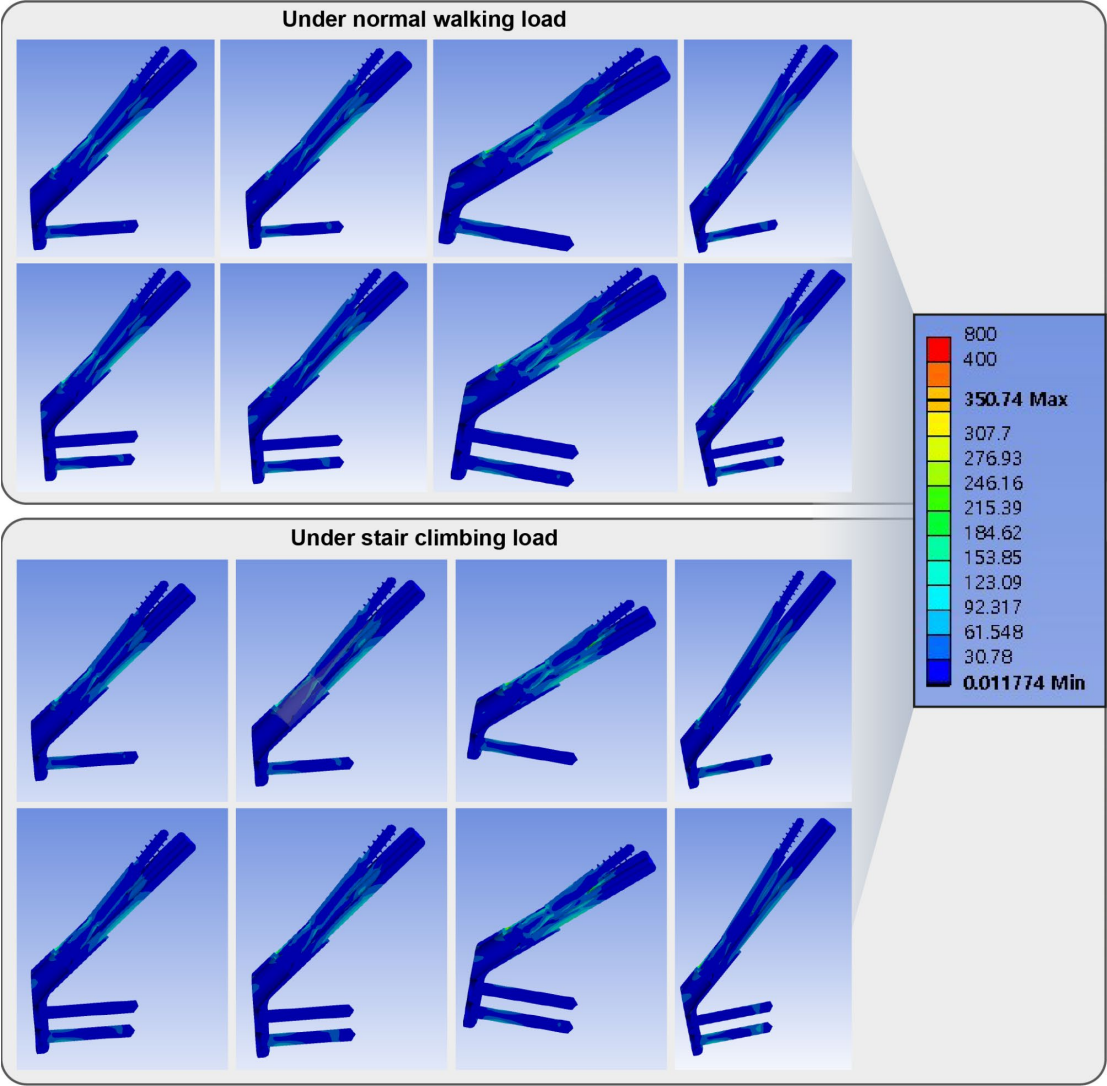
Band graphs depicting von Mises stress of the implant in the normal walking (upper eight graphs) and stair climbing (lower eight graphs) conditions. The graphs within each loading condition were arranged in the same sequence as in Fig. 1. All graphs share the color legend

Peak VMS of implants ranged from 214 to 351 MPa. The highest peak VMS was still lesser than the yield strength of the titanium alloy. (Fig. 6; Table 2)

**Fig. 7:**
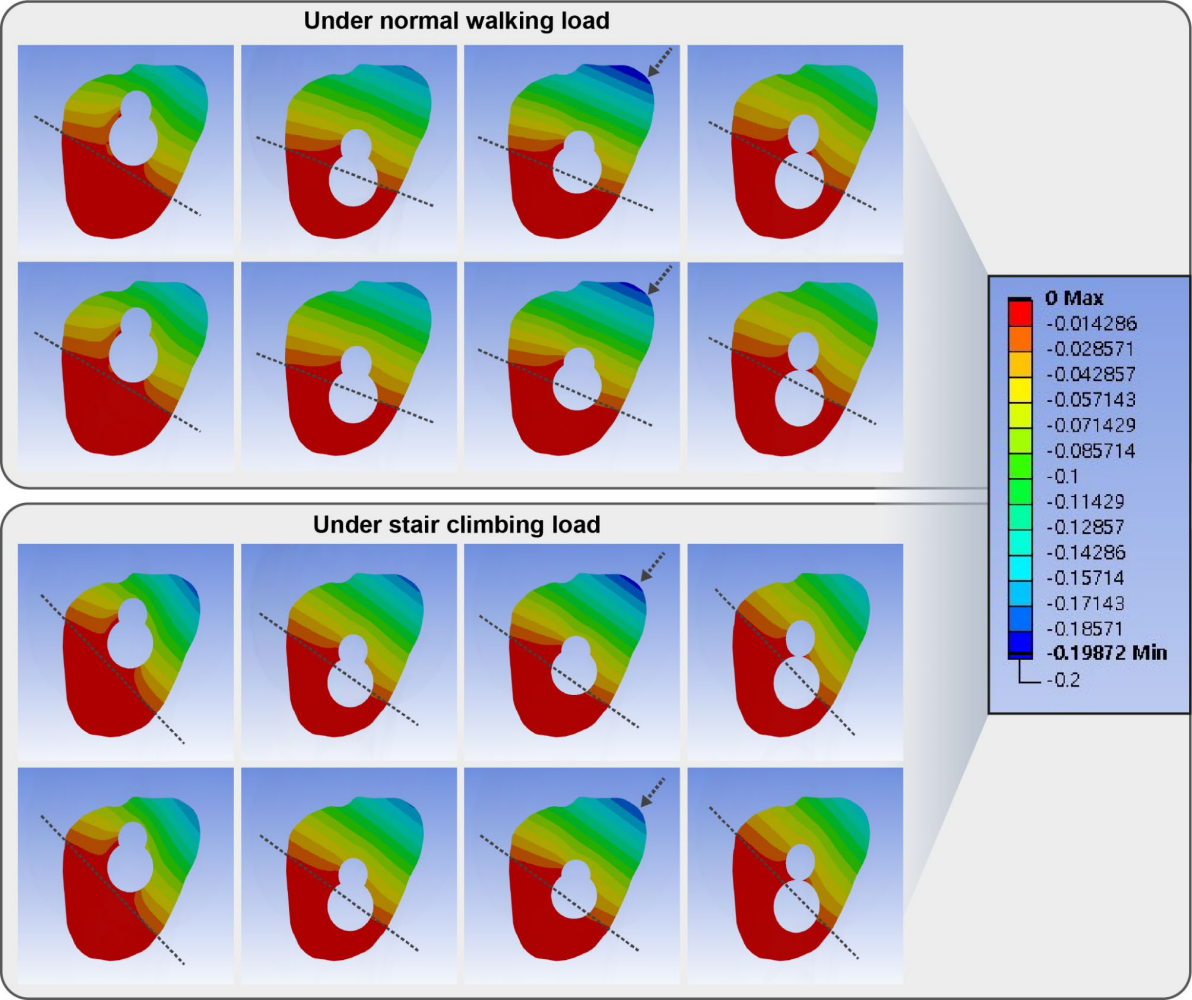
Band graphs depicting gaps between fracture surfaces in the normal walking (upper eight graphs) and stair climbing (lower eight graphs) conditions. The graphs within each condition were arranged in the same sequence as in Fig. 1. All graphs share the color legend. Eccentric loading on bent tube geometry of femur causes rotational moment on proximal fragment with implant as rotational center. The surface medial to implant results in compression, while the surface lateral to implant results in tension and gap. Bolt trajectory determines boundary between compression and gap. The trajectory of the bolt determines the penetration point of the implant through the fracture surface, and the penetration point determines the boundary between the region experiencing compression and tension. Maximum gap is formed at the anterosuperior surface in all models (Black arrow). The pattern of interfragmentary gap is not affected by the length of plate while the pattern is majorly affected majorly by the trajectory (dashed lines). The gap in varus trajectory models is 120% of the gap in central trajectory models under normal walking load and 107% in stair climbing load. The gap in valgus trajectory models is 87–88% of the gap in central trajectory models under normal walking load and 87% in stair climbing load

**Fig. 8:**
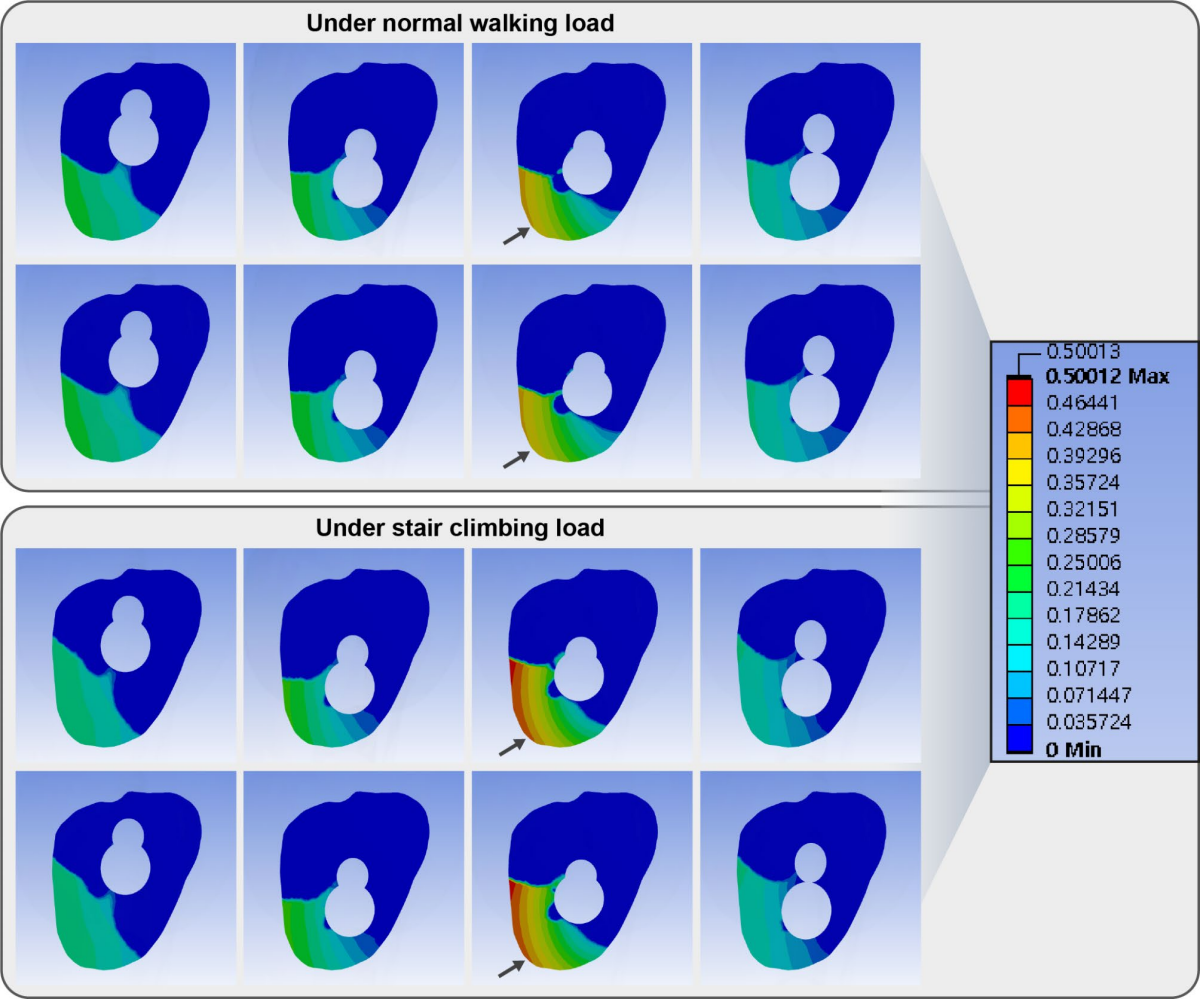
Band graphs depicting sliding distances in the normal walking (upper eight graphs) and stair climbing (lower eight graphs) conditions. The graphs within each condition were arranged in the same sequence as in Fig. 1. All graphs share the color legend. While the anterosuperior surface have interfragmentary gap, the posteroinferior surface with compression experience interfragmentary sliding to make shear stress on the tissue between both fragments. Although central, inferior, and valgus trajectories had similar pattern of sliding, varus trajectory had exceptionally larger (180% of the sliding distance of central trajectory in the normal walking load, 240% of the sliding distance of central trajectory in the stair-climbing load, Black arrow)

The inferior trajectory had a comparable gap and 10% more sliding between fracture fragments than the central trajectory (Figs. 7 and 8; Table 2). Varus trajectory had 20% greater gap and 80–82% more sliding between fracture fragments than that of the central trajectory. In contrast, valgus trajectory had 14% less gap and 18% less sliding distance than the central trajectory. The difference in gap and sliding distance between 1-hole and 2-hole plates was < 5% in all trajectories of the bolt.

### Stair-climbing condition

The trabecular bone located in the narrow cleft between the anti-rotation screw or under the plate which supports the bolt had the absolute magnitude of strain over 1% as well under the stair climbing load. (Figures 4 and 5)

In the models with bolts in the inferior trajectories, the endosteal cortex in the inferior neck supporting the bolt had − 1.33% (model with the bolt in the inferior trajectory and 1-hole plate) and − 1.34% (model with the bolt in the inferior trajectory and 2-hole plate) of the peak minimum principal strain. (Fig. 5; Table 2)

The screw-holding cortical bone in subtrochanter in the model with 2-hole plate and the bolt in inferior trajectory and in the models with 1-hole or 2-hole plate and the bolt in valgus trajectory had shown 1.49%, 1.17% and 1.25% of maximum principal strain, respectively. (Fig. 4; Table 3)

Peak VMS of implants ranged from 259 to 424 MPa. The highest peak VMS was still lesser than the yield strength of the titanium alloy (Fig. 6; Table 2).

The inferior trajectory had a comparable gap and about 30% more sliding between fracture fragments than the central trajectory (Figs. 7 and 8; Table 2). Varus trajectory had less than 10% grater gap and 138–140% more sliding between fracture fragments than that of the central trajectory. In contrast, valgus trajectory had 13% less gap and 5% less sliding distance than the central trajectory. The difference in gap and sliding distance between 1-hole and 2-hole plates was < 5% in all trajectories of the bolt.

## Discussion

The present study evaluated the mechanical effects of the length of plate and the trajectory of bolt of FNS on the stability of Pauwels type III femoral neck fractures and strain around distal screw. The stability of fracture surface was more dependent on the trajectory of the bolt than on the length of the side plate. Although the strain of cortical bone around the most distal locking screw was affected by both the trajectory of the bolt and the length of the side plate, the longer plate consistently resulted in greater strain than the shorter plate. While the bolt in the valgus trajectory provides more stability on the fracture surface compared with bolts in the other trajectories, strain of cortical bone around locking screw was greater compared to those of the bolt in the central trajectory.

With structural similarities of sliding mechanism, the study on the effect of the proximal femoral nail antirotation blade trajectory on fracture stability was in line with the present study results [32]. In the previous finite element analysis, the larger area above the implant penetration was, the lesser stability at the fracture site was expected. Similarly, the present study also indicated that the inferior trajectory of the bolt had a larger area of fracture surface above the bolt compared with the central trajectory of the bolt, resulting in more fracture gap and sliding. As the component vector of the load perpendicular to the bolt axis acts as a shear force for the fracture surface, this affects the imbalance of the surface contact [32]. The varus trajectory has a larger angular difference between the load and bolt axis than that of the central trajectory, while the valgus trajectory makes the angular difference smaller. Thus, the trajectory determines the biomechanical environment at the fracture interface and the area under tensile load by a combination of the angular difference, load, and the penetration area of implant on the fracture surface.

The selection of the number of holes on the side plate is an unavoidable step during the fixation with FNS; however, the decision is rarely made based on the knowledge of biomechanical effects on the behavior of fixed fracture. The results of the present study indicate that while the 2-hole plate of FNS consistently decrease the interfragmentary gap under both normal walking and stair climbing load, the influence was limited compared to the change from bolt trajectory. The differences of sliding distance were merely affected by the length of plate staying within 1% changes.

While the improvement of fracture stability made by the 2-hole plate was limited, the longer plate even posed more risks of failure of cortical bone around distal locking screw. Although dynamic hip screw (DHS) is also an extramedullary fixation device with fixed angle similar to FNS, the studies on the DHS reported that very short plates cannot play their role adequately contrast to the present study [15, 33]. This might be due to the differences in the fracture geometry which both devices are used for. The bolt of the FNS has to get through a longer corridor of trochanteric bone to reach the fracture surface in femoral neck fractures than the lag screw of the DHS does in trochanteric fractures. Recently, one finite element analysis with femoral neck fracture of extreme Pauwels angle reported the different stiffness between neck-fractured femurs fixed with 1-hole and 2-hole plates [12]. The study assumed that the femur model of a 26-year-old male patient was made with two kinds of materials, which were homogenous cortical and trabecular bone, with the femoral head and trochanter wrapped with thick cortical bone layer. The deformation of fracture model was provided to evaluate the stability of fracture. The deformation of the fractured femur fixed with implants results not only from the stability of the fracture surface but also from the stiffness of the implant. Only the joint reaction force was assumed for the loading conditions. In our study, the Pauwels angle was 60°, and 150 material properties were assigned according to the greyscale of the matched voxel without any geometrical modification of the femur. The comparative parameters included maximum and minimum principal strain, VMS of implant, and interfragmentary gap and sliding distance. The loading condition included multiple muscle contractions as well as the joint reaction force. Despite different boundary conditions, our study aligns with Fan et al. who found no stability difference between 1-hole and 2-hole plates in Pauwels angle 60° fractures, still classified as Pauwels type III. Fan et al. recommended 2-hole plates for Pauwels angle > 70°, but our study suggests that altering bolt trajectory has a greater impact on stability than using a 2-hole plate.

In contrast to the expectation that the 2-hole plate would provide more stability on fracture fixation than the 1-hole plate, the present study indicated that 2-hole plate did not provide more stability to the fracture surface but provided more strain on the cortical bone around the distal-most screw.

Although subtrochanteric fracture after FNS fixation is reported occasionally, the risk factor of the devastating complication is yet to be clarified [34].

With a long history with multiple cannulated screw fixation for femur neck fractures, starting the distal-most screw distal to the lesser trochanter has been accused of subtrochanteric fracture [35]. Although a biomechanical study reported that the placement of drill holes along the screw trajectory on cadaveric femurs did not pose a risk of subtrochanteric fracture, it nevertheless casts doubt on the assumption that the position of the distal-most screw affects the risk of subtrochanteric fracture [36]. The seemingly conflicting results may be due to whether or not the metal implant crosses the endosteal corridor of the femoral neck, as this can affect the transfer of the mechanical load from the femoral head to the lateral cortex of the femur and thus play a crucial role in subtrochanteric fracture. Combining the results of the present finite element analysis and the similarity in the mechanical role of the FNS bolt to the cannulated screw in femoral neck fracture, the use of longer plate in FNS only positions the distal-most screw more distally, risking subtrochanteric fracture without a benefit in the stability on the fracture surface.

Without validation experiments, the absolute values in the present study do not precisely reflect the outcomes in real clinical settings. Therefore, it is essential to interpret the data primarily within the context of the comparisons made among the models provided. The absolute values from the literature would aid clinicians in figuring out the clinical relevance. Assuming the yield strain of the bone to be 1%, models with peak maximum or minimum principal strain more than 1% have risks of cortical failure [37].

Although interesting results can be drawn from our model, this study has some limitations. As finite element analysis uses varied assumptions for simplification, there would be concerns if our findings correlate to clinical results.

The femur models were generated from the CT images of an older patient, and materials were assigned properties according to the respective element based on the grey values of the matched voxels. Although the finite element model of the femur was mapped from the CT HU of an older patient who had trochanteric fracture of the contralateral femur, the properties of the bone were assumed to be isotropic and elastic. The assignment of material properties based on the CT HU may assist in closing the gap between simulations and the real-world. Various factors, such as patient-related, surgeon-related, and fracture-related factors, were known to affect the outcome of the fracture treatment. As we focused on the specification of FNS in the present analysis, all other surgical targets, such as reduction of fractures, were assumed to be complete, and there was no gap at the fracture interface before loading was assumed. We believe that the qualitative insights drawn from the present comparative analysis would deepen the understanding of FNS and further experimental or clinical study based on the insights would deepen the understanding of the accurate mechanics of FNS.

## Conclusion

For the fixation of Pauwels type III femoral neck fracture, the trajectory of the FNS bolt mainly affects the mechanical stability of the fracture and the length of plate mainly affects the strain of cortical bone around the distal-most screw. While varus and inferior trajectories of the bolt affected the stability of the fracture negatively, valgus trajectory of the bolt had risks of excessive strain in subtrochanteric cortex. The 2-hole plate cannot compensate for the malposition of the bolt trajectory and only increases the risk of excessive strain in subtrochanteric cortex. The surgical target should stay on the central trajectory of the bolt and 2-hole plate did not have mechanical benefits exceeding the risk. The conclusion of the present FEA should be cautiously inferred and requires further biomechanical and clinical analysis for confirmation.

## Data Availability

The datasets generated and/or analyzed during the current study are not publicly available, but are available from the corresponding author on reasonable request.

## Declarations

**Ethics approval and consent to participate**.

The requirement for informed consent was waived, and the study protocol was approved by the institutional review board (IRB) of Ajou university hospital (IRB number: AJIRB-MED-MDB-21-696). All methods in the study were carried out in accordance with the Helsinki Guidelines and Declaration.

## Abbreviations

FNS: Femoral neck system
DHS: Dynamic hip screw
CT HU: Computed tomography Hounsfield unit
VMS: Von Mises stress
